# AI-Driven Tuberculosis Hotspot Mapping to Optimize Active Case-Finding: Implementing the Epi-Control Platform in Uganda

**DOI:** 10.3390/tropicalmed11020036

**Published:** 2026-01-28

**Authors:** Geofrey Amanya, Sumbul Hashmi, Jessica Sarah Stow, Philip Tumwesigye, Bernadette Nkhata, Kelvin Roland Mubiru, Anne-Laure Budts, Matthys Gerhardus Potgieter, Seyoum Dejene Balcha, Muzamiru Bamuloba, Andiswa Zitho, Luzze Henry, Mary G. Nabukenya-Mudiope, Caroline Van Cauwelaert

**Affiliations:** 1 Ministry of Health Uganda, Lourdel Road, Plot 6, Lourdel Road, Nakasero, Kampala P.O. Box 7272, Uganda; 2 EPCON, Lange Gasthuisstraat 29/31, B-2000 Antwerp, Belgium; 3 EPCON, Workshop 17, 146 Campground Rd, 3rd Floor, Snakepit Building, Newlands, Cape Town 7780, South Africa; 4 LPHS-TB and Urban Health Activity, Infectious Diseases Institute, College of Health Sciences, Makerere University, Kampala P.O. Box 22418, Uganda

**Keywords:** hotspots, tuberculosis, active case-finding, artificial intelligence

## Abstract

Tuberculosis remains a major public health concern in Uganda, one among the thirty high TB burden countries globally. Despite national progress, gaps persist due to asymptomatic disease, diagnostic limitations, and uneven access to healthcare within the country. This study implemented the Epi-control platform, an AI-driven predictive modelling tool, to predict community-level hotspots and support data-driven active case-finding (ACF). Using retrospective chest X-ray screening data, we integrated demographic, environmental, and human development indicators from open-source databases to model TB risk at sub-parish level. A proprietary Bayesian modelling framework was deployed and validated by comparing TB yields between predicted hotspots and non-hotspot locations. Across Uganda, the model identified significantly higher TB yields in hotspot areas (risk ratio = 1.69, 95% CI 1.41–2.02; *p* < 0.001). The Central and Western regions showed the highest concentrations of hotspots, consistent with their population density and urbanization patterns. The results show that the model prioritized areas with higher observed ACF yield in this retrospective dataset, supporting its potential operational use for screening prioritization under similar implementation conditions. The results demonstrate that AI-based predictive modelling can enhance the efficiency of ACF by targeting high-risk areas for screening. Integrating such predictive tools within national TB programmes may support screening planning and resource prioritization; prospective evaluation and external validation are needed to assess generalisability and incremental impact.

## 1. Introduction

Tuberculosis (TB) continues to pose a significant public health challenge in Uganda. The country is among the thirty high TB burden countries globally, with an estimated 240 new cases reported daily [1]. While stakeholders use national estimates of incidence and prevalence to determine high-burden areas, these figures can vary locally due to differences in local risk factors [2]. Moreover, passive case-finding often fails to fully capture the true TB incidence [3,4]. This is attributable to factors such as limited healthcare access, asymptomatic cases, diagnostic shortcomings, or misdiagnosis as other respiratory ailments [4]. Active case-finding (ACF) proactively identifies individuals at risk of TB, a shift from reactive healthcare [3,5]. It is vital for diagnosing TB in both urban and rural areas, stopping its spread through early detection and treatment [6]. ACF also helps to understand challenges faced by individuals that prevent them from seeking care, allowing efficient resource allocation and reducing disease burden [7].

Ugandan studies highlight the effectiveness of ACF strategies in boosting TB case detection. One study found targeted community-based ACF had a tenfold higher screening yield than healthcare facility screening (3.7% vs. 0.3%); this targeted screening included yield from contact tracing and screening in slums, markets, and taxi/bus terminals [8]. Another study by Robsky et al. in Kampala showed screening 20% of the population could find 40% of undiagnosed TB cases [7]. Aceng observed a rise in TB case notifications in Uganda, especially after ACF implementation began in 2018 [2]. Given that ACF requires significant resources, it is essential to implement data-driven and evidence-based strategies to enhance systematic and cost-effective screening. Methods like GIS-based hotspot mapping [4,7,9,10] effectively target resources to TB hyperendemic zones or hotspots. Traditional geospatial mapping methodologies predominantly rely on routine notification data, thereby limiting their accuracy to the quality of the data itself. In countries where notification data fails to encompass all instances, it becomes imperative to explore alternative and innovative strategies.

Leveraging the growing adoption of AI and machine learning approaches, we implemented a proprietary solution from EPCON, called the ‘Epi-control platform’ to identify community-level TB hotspots in Uganda. The solution has previously been implemented in Pakistan [11], Nigeria [12], Central African Republic [13], and The Philippines to mention a few, in collaboration with local partner organizations and the National TB control programmes.

The Epi-control platform is a software, accessible via a browser, which uses AI to strengthen public health. The platform uses principles of machine learning to develop an epidemiological digital representation (“twin model”) of geospatial TB positivity across Uganda. This digital twin model was based on data generated from local ACF programme implementation and contextual data, following the principles of the MATCH framework [14,15]. The outputs were visualized on a customized Geoportal with an intuitive interface. The local stakeholders can access the platform for routine planning of community outreach interventions for the wider general population, assessing the distribution of healthcare services and countrywide hotspot predictions down to the community level.

This study aims to assess the ability of the Epi-control platform to effectively predict ‘hotspots’ that could be targeted for improving ACF outcomes in Uganda.

We intend to achieve the following objectives:

- Use bacteriological confirmed TB positivity rate from ACF interventions along with local contextual data to predict TB positivity rate at sub-parish level across the four regions.
- Map the predicted output, the bacteriological confirmed TB positivity rate on a customized geoportal for geospatial visualization and local stakeholder engagement in Uganda.
- Estimate the difference in yield (bacteriological confirmed TB cases/screened) by comparing predicted ‘hotspots’ with ‘non-hotspots.’

## 2. Materials and Methods

### 2.1. Study Design and Setting

A retrospective observational study was conducted on the data generated from community-based ACF interventions implemented under the United States Government (USG)-funded Local Partner Health Services for Tuberculosis (LPHS-TB) activity, led by the Infectious Diseases Institute (IDI) in collaboration with other implementing partners. The project data was collected between 30 January 2023 to 7 October 2025. The programme implementation involved national, subnational, and community stakeholders. The National Tuberculosis and Leprosy Control Program (NTLP) provided strategic oversight, while district, regional, and community civil society organizations managed demand creation, mobilization, logistical support for mobile services (van, digital X-rays), and patient linkage to care. Additional partners included USG-supported grants and The Global Fund.

ACF activities implemented multi-pronged interventions in 15 sub-regions (146 districts) across Uganda. These interventions included verbal screening for symptoms, mobile digital chest X-ray (DCXR), and CXR vans equipped with CAD (Computer Aided Diagnostics) for TB screening to identify presumptives, followed by GeneXpert testing for bacteriological confirmation, all in adherence with the national screening algorithm. At community level, the project engaged community-based civil society organizations (CSOs) and village health teams volunteers (VHTs) for contact tracing of index TB cases enrolled for treatment, community outreach, active case-finding, and sensitization meetings in the communities. This diverse coverage up to the community level ensured the model was trained on data from varied epidemiological and socio-economic contexts.

### 2.2. Inclusion and Exclusion Criteria

High-risk groups such as individuals in prisons, diabetic clinics, or HIV outpatient departments were screened at designated sites. However, for the purposes of our predictive modelling, we excluded data from these high-risk subgroups. This decision was made because their potentially higher yield could skew the results, making it difficult to accurately assess TB transmission patterns within the broader general community. Sub-parishes with a screening count below 25 individuals were excluded from the analysis to mitigate potential bias arising from low statistical power, which could result in unreliable yield estimates. Consequently, a total of 31 sub-parishes were excluded: 1 from the Northern region, 8 from the Western region, 13 from the Central region, and 9 from the Eastern region.

The ACF interventions aimed at the broader general population were conducted through community-based screening events, where all individuals regardless of risk factors or symptoms had the opportunity to be screened. While the intervention included both verbal- and X-ray-based screening to identify presumptive TB followed by bacteriological confirmation, we chose to use only chest X-ray-based screening and bacteriological confirmed cases from therein for modelling TB hotspots. Chest X-rays have higher sensitivity compared with verbal screening and can detect asymptomatic TB [16,17]; the final dataset could provide a better understanding of the actual distribution of undiagnosed TB in the community. This meant that we included 26% of the total data available from the screening intervention. Throughout this paper, Chest X-ray-based ACF data/events refer to individuals in the general community screened with a chest X-ray device followed by bacteriological confirmation with GeneXpert test. This includes all chest X-ray screenings, with and without verbal screening and both computer aided and human readings.

### 2.3. Input and Outcome Variables

The TB positivity rate was defined as the proportion of bacteriological confirmed TB cases from the total individuals screened by chest X-ray devices (observed yield), serving as the model training variable. Predicted TB positivity rate was the outcome of interest.

### 2.4. Covariates

Data regarding sociodemographic and human development indicators, recognized as factors linked to TB [18,19], were obtained from open-source platforms. The total population, encompassing females, males, and the elderly, was sourced from Facebook [20]. Population density was calculated as the number of people per square kilometre for each unit. Data on poverty, distance to major roads, were obtained from WorldPop [21]. Data on childhood vaccinations, including the full 8 basic vaccinations, DPT1 and 2, and the measles vaccine were obtained from spatially modelled data from Demographic and Health Surveys (DHS) [22]. Data on male and female literacy [23], childhood nutrition (percentage of underweight children) [24], and mortality under 5 [25] were obtained from Institute for Health Metrics and Evaluation (IHME). Access to water, sanitation availability [26], HIV prevalence [27], night-time lights [28], elevations [21], Gridded Relative Deprivation Index (GRDI) [29], and motorized travel time to a health facility [30] were also included in the assessment. Table 1 lists the variables, and more details are available in Table A1 in the Appendix A.

### 2.5. Resolution, Data Preparation, and Model Training

Uganda consists of four major regions: namely the Central, Eastern, Northern, and Western regions. These are further divided into 15 sub-regions referred to as health regions, which sub-divide into 146 districts, 312 counties, 2208 sub-counties, and 10,864 parishes. The parishes were further divided into smaller units called ‘sub-parishes’ by using the K-means clustering algorithm such that each sub-parish contained a population of approximately 10,000. The approach has been explained in more detail in another publication [12,14].

All variables listed in Table 1 were aggregated to the sub-parish level. They were then scaled to a rate form, providing each unit with a unique profile based on its local sociodemographic contextual information. Sub-parishes with a screening count lower than 25 were excluded from the analyses and modelling.

Chest X-ray-based ACF events were organized across 10,864 sub-parishes, the geo-coordinates of each event were shared by the implementing partners that enabled precise mapping. The model was trained on the observed sub-parish TB positivity rate to predict a TB positivity rate for all sub-parishes including those with observed data. The predicted output thus allowed for identification of other areas that could be prioritized for ACF activities.

### 2.6. Modelling Approach

We used a probabilistic Bayesian network regression model to estimate sub-parish TB positivity rates from observed chest X-ray screening outcomes and contextual covariates. The model represents the joint distribution of the target and predictors as a directed acyclic graph and is used here as a predictive model; it does not, on its own, support causal inference.

Candidate predictors were pre-specified from established determinants of TB transmission and access to care and comprised the contextual indicators listed in Table 1. Bayesian network structure was learned with causal-learn python package using the Peter-Clark (PC) algorithm for causal discovery with the Fisher-Z test for conditional independence and an alpha sensitivity setting of 0.9. A maximum parent cutoff of 5 parents to the target node were used, and nodes not directly connected to the target variable were pruned. The pgmpy python package [31] was used to fit linear Gaussian conditional probability distributions (CPDs) for parameter estimation were fitted using Maximum Likelihood Estimation (MLE). No informative Bayesian priors were specified in this analysis; parameters were estimated via MLE. Model inference was performed using propagation of evidence through the network’s linear Gaussian conditional probability distributions using pgmpy.

### 2.7. Model Evaluation and Comparison

To evaluate predictive performance, a fivefold cross-validation approach was applied across all sub-parishes in the country. The data were first partitioned into five groups (folds) using the GroupKFold method. For each iteration, four folds (80% of the data) were used to train a Bayesian model, while the remaining fold (20%) served as the validation set to test predictive accuracy. This process was repeated five times so that every sub-parish was used once for validation. Model accuracy and calibration were then assessed by comparing predicted TB positivity rates against the observed rates in the held-out folds.

To interpret hotspot performance, predictions were sorted by estimated positivity rate, and the top 30% of sub-parishes were classified as hotspots, and the remainder as non-hotspots, based on prior cross-country calibration. This 30% cut-off was selected a priori as an operational planning parameter, based on prior deployments and previously published work in Nigeria [12], where prioritizing approximately the top 30% of areas provided a pragmatic balance between concentrating screening resources and maintaining sufficient geographic coverage for implementation. We note that the hotspot threshold is a tuneable programme decision (rather than an intrinsic property of the model) and may be adapted to local screening capacity and coverage objectives; alternative thresholds such as 10%, 20%, or 40% could be used in other contexts. Based on the combined outputs across folds, relative yield metrics were computed: hotspot yield vs. non-hotspot yield (positives per screened), risk ratio, relative risk increase (RRI), risk difference, number needed to screen (NNS), and statistical significance via Fisher’s Exact Test.

Additionally, a stratified evaluation was conducted to assess model performance across the four major regions, to evaluate whether the predictive accuracy was consistent across distinct epidemiological contexts. Hotspots were identified based on the top 30% of predicted TB positivity rates, with the remaining 70% classified as non-hotspots.

Because observations are aggregated at the sub-parish level, neighbouring sub-parishes may exhibit spatial autocorrelation in both screening yield and predicted positivity. We did not explicitly adjust for spatial clustering in this internal validation; therefore, performance estimates and inferential comparisons should be interpreted with this dependence in mind.

## 3. Results

We analyzed chest X-ray screened ACF data aggregated across four regions: Central, Eastern, Northern, and Western. The four regions were subdivided into 146 districts, which were further divided into 10,864 parishes. Key metrics presented for each region are presented in Table 2. Across the country, a total of 33,427 individuals were screened using chest X-ray devices, of which 465 were diagnosed with bacteriological confirmed TB. Screening data was available for 3.63% of sub-parishes in the Central region and 1.21% in the Northern region.

The distribution of the number and proportion of hotspots and non-hotspots across the country by region are illustrated in Table 3. The Central region was subdivided into 1958 sub-parishes, of which 48.7% met the 30% cut-off, thus categorized as hotspots. The Western region had 2915 sub-parishes, of which 42.8% were categorized as hotspots.

We compared the difference in TB case-finding yield in predicted hotspots with non-hotspots, as shown in Table 4. Across the country, chest X-ray screening identified 215 TB positive individuals in hotspot areas (from 11,279 screened) and 250 in non-hotspot areas (from 22,148 screened), corresponding to a risk ratio of 1.69 (95% CI: 1.41–2.02; *p* < 0.001). At the regional level, risk ratios ranged from 1.52 (95% CI: 1.10–2.10; *p* = 0.011) in the Eastern region to 2.18 (95% CI: 1.32–3.60; *p* = 0.004) in the Central region. Statistically significant associations were observed in all four regions.

**Figure 1:**
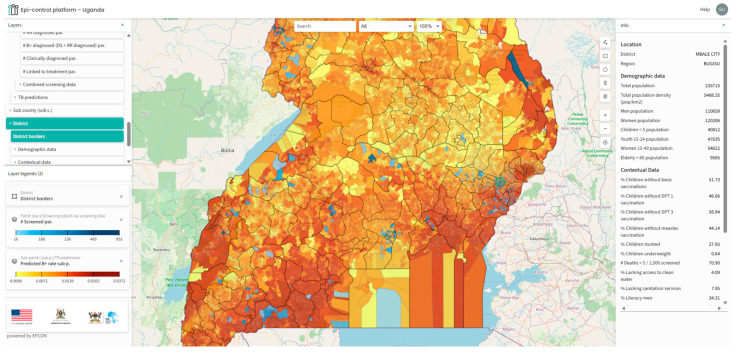
Visualization of predicted TB positivity rate on the Epi-control Geoportal at sub-parish level. Light yellow represents lower rate and dark red represents higher rate. Interspersed blue areas (in-land) represent sub-parishes where observed data was present (light blue to dark blue). District borders are represented with black lines.

We mapped the predicted outputs on a customized geoportal for easy access for the stakeholders (Figure 1). The geoportal provides a range of functionalities, including hotspot filtering and integration with Google Maps, enabling local teams to effectively plan community-based interventions. Additionally, the use of a choropleth map facilitates more intuitive interpretation of results for users without technical expertise.

## 4. Discussion

This study presents an internal validation of a predictive hotspot mapping approach (the Epi-control platform) applied to Uganda. The platform uses a Bayesian network regression model that integrates ACF outcomes with contextual covariates to estimate and rank sub-parish-level TB positivity and is used here for prediction rather than causal inference. We compared the difference in TB case-finding yield in predicted hotspots with non-hotspots.

The model predicted a variable number of hotspots across regions, and hotspot areas showed higher observed ACF yield than non-hotspots in internal validation, indicating that the model concentrates observed yield under similar screening processes.

An analysis of the predicted hotspot distribution reveals that the highest number of hotspots were identified in the Central region (48.7%), followed by the Western regions (42.8%). Conversely, the Northern and Eastern regions exhibited the fewest predicted hotspots.

The Central region demonstrated the highest screening coverage, succeeded by the Eastern, Western, and Northern regions, respectively. According to Uganda’s 2002 census, the Central region accounted for 27% of the country’s population, the Western region 26%, the Eastern region 25%, and the Northern region 22%. Furthermore, the Central region contained 54% of the urban population (predominantly in Kampala), while the Northern, Western, and Eastern regions accounted for 17%, 14%, and 13% of the urban population, respectively [32]. Given the higher population density and urban settlements in the Central and Western regions, the model’s prediction of a greater proportion of hotspots in these areas aligns with the typical distribution of TB transmission, which tends to be higher in populous urban settings [33,34].

Henry et al. developed a spatial model utilizing TB prevalence survey data from 2014 to 2015, TB notification data from 2016 to 2019, and additional contextual covariates such as population estimates, household crowding, night-time light intensity, and travel time to health facilities, among others, to generate district-level TB estimates [4]. The geographic distribution produced by this model indicates that most high-burden districts were situated in the Northern and Central regions of the country, with some also identified in the Western region. Our model demonstrates that most hotspots are concentrated in the Central and Western regions, with several present in the east. Although there are certain similarities between the maps generated from both studies, direct comparison is not feasible due to differences in resolution (district versus sub-parish level), data sources, and modelling methodologies. The difference could also be attributed to the ACF data included in our analysis, we had the most extensive screening coverage in the Central region and the least in the Northern region. With ongoing programme implementation and updates to the dataset, the model’s outputs may undergo further refinement over time. Variations in the definition of hotspots can also lead to relative differences.

Aceng et al. conducted a study mapping district-level TB notifications over a ten-year period (2013–2022) [2]. Their findings reveal that Moroto and Napak districts in the north, as well as Kampala and Kalangala districts in the Central region, consistently reported high notification rates over several years. Our model’s results align with these observations, predicting multiple hotspots in these districts at the sub-parish level. Aceng et al.’s maps consistently show low notifications in Mbale and Soroti districts, this could be due to low reporting, inadequate screening, or genuinely low TB transmission; our model predicts Mbale district as a potential hotspot. Our ACF dataset included high screening coverage in this region together with the highest number of confirmed TB cases in the Eastern region. This highlighted an advantage of utilizing community-based active case-finding (ACF) data over notification data for identifying TB hotspots. By learning from both community-based interventions and local contextual data, the model facilitates a more comprehensive understanding of the population’s local health situations.

A significant burden of TB was predicted in large areas of the Western region (Table 1 and Figure 1). While some studies suggest the burden in this region is below the national average [4], our finding may alternatively indicate a higher prevalence of undetected TB, which is not captured by notification-based studies. Wynne et al. reported that the National TB control programme in the Western Uganda region encounters substantial challenges, with the healthcare system facing financial constraints that adversely affect timely diagnosis and patient outcomes [35]. The Western region shares borders with the Democratic Republic of Congo (DRC), Tanzania, Rwanda, and Burundi, potentially resulting in a high migrant population. This demographic group frequently experiences financial hardship, difficult living conditions, and systemic barriers to accessing and maintaining continuous care [36,37]. It is plausible that the model identified a potentially underserved region with an elevated risk of undiagnosed TB.

Based on Fisher’s Exact Test and relative yield metrics, ACF yield was 68% higher in predicted hotspots than non-hotspots in this internal validation, indicating that the model concentrates observed yield under the same screening dataset. In practical terms, for every 100 TB cases identified through routine screening methods, employing the Epi-control platform could lead to the identification of approximately 168 cases under similar conditions. Although ACF interventions are costly, targeting predicted hotspots may improve operational efficiency by prioritizing areas with higher observed yield per screened; cost-effectiveness and incremental impact require prospective study.

The geoportal developed to visualize predicted hotspots supports operational use of the model outputs by enabling stakeholders to review prioritized areas and associated contextual layers. The platform enhances the applicability of predictive outputs by allowing for targeted interventions in specific communities, which can lead to improved TB case notifications and optimized resource allocation. The portal also visualizes contextual data layers for further analysis and epidemiological insight.

This evaluation constitutes internal validation because both model training and evaluation are based on the ACF screening dataset. Accordingly, the hotspot–yield comparison should be interpreted as a test of whether the model concentrates observed yield under similar screening processes, rather than as evidence of causal effects.

Our approach had certain limitations and potential biases. Primarily, selection bias may have occurred because we relied on ACF screening data. This data includes individuals reached through organized outreach events, possibly underrepresenting remote, underserved, or stigmatized populations. In addition, ACF implementation is not uniform in space or time; therefore, predicted hotspots may partly reflect operational deployment patterns and screening intensity (e.g., where mobile units were deployed more frequently or where ACF activities were already concentrated or yielded higher detection), rather than underlying TB risk alone. While we included several contextual indicators to capture broader determinants of TB risk and access to care, residual selection and implementation bias may remain. In addition, spatial clustering of risk and of ACF operational deployment may induce correlation between nearby sub-parishes; we did not incorporate spatial random effects or spatially blocked resampling in the current analysis. As a result, statistical significance tests comparing hotspots versus non-hotspots may be overconfident, and we emphasize effect sizes (e.g., risk ratios) as descriptive measures within this internal validation framework. Although efforts were made to incorporate the most recent ACF data, obtaining high-resolution contextual data proved challenging. As a result, some datasets utilized in this model are more than five years old. While the use of older data may introduce uncertainties in the results, we are confident that the benefits of producing highly granular estimates outweigh the potential drawbacks associated with these data limitations. A wide range of data sources were integrated; however, certain potentially valuable datasets, such as those concerning actual notifications, prevalence survey, migration patterns, were unavailable at the requisite resolution. We consider these data sources to be highly beneficial for future modelling efforts and for assessing generalisability and incremental impact.

Utilizing ACF data for predictive modelling also offered a significant advantage. It allowed the model to learn the distribution of undiagnosed TB cases within the community, thereby potentially enhancing its ability to predict new ACF sites more effectively than facility-level notification data alone [12]. This approach minimizes the dependence on notification data and facilitates data-driven decision-making in low-resource environments.

This approach, a first for Uganda, involved predicting subnational TB hotspots at a sub-parish level. Therefore, it is hard to validate the predicted geographical distribution of high TB burden areas by comparing with existing evidence. However, studies that highlight the potential challenges or drivers of TB transmission in specific regions helped us understand whether the predicted distribution aligns with the available knowledge. The test of significance comparing yield in the hotspots with non-hotspots is therefore a satisfactory approach for a quantitative comparison. Overall, the platform showed internal validity in concentrating observed ACF yield in predicted hotspots and may support screening prioritization; external validation and prospective implementation studies are needed to assess generalisability and incremental impact.

## Figures and Tables

**Table 1:** Local contextual data from open sources to inform subnational burden estimation.

Indicator	Source	Year
Overall population density Elderly (ages 60+) population Female population Male population	Facebook’s Connectivity Lab and the Center for International Earth Science Information Network	2016
Distance to major Roads Elevation	WorldPop	2018
% children with DPT-1, DPT-3, Measles vaccination Fully vaccinated (8 basic antigens)	Uganda Bureau of Statistics (UBOS) and ICF	2018
Under 5 (0–5 years old) mortality probability Access to any improved water source Reliance on open defecation HIV prevalence Literacy in male and females Percentage of children underweight	Institute For Health Metrics and Evaluation	2019
Motorized travel time to health facility	Malaria Atlas Project (MAPS)	2019
Nightlights (nanoWatts/cm^2^/sr)	Earth Observation Group	2023
Relative Deprivation Index	NASA Socioeconomic Data and Applications Centre	2020

**Table 2:** Chest X-ray screened ACF data and descriptive statistics across the country.

Region	Metrics	N
Country	Total parishes	10,864
	Total Sub-parishes	11,153
	Sub-parishes screened	243
	Screening coverage	2.18%
	Total Individuals screened	33,427
	TB diagnosed	465
Central Region	Total parishes	1765
	Total Sub-parishes	1958
	Sub-parishes screened	71
	Screening coverage	3.63%
	Total Individuals screened	11,322
	TB diagnosed	111
Eastern Region	Total parishes	3599
	Total Sub-parishes	3626
	Sub-parishes screened	98
	Screening coverage	2.70%
	Total Individuals screened	10,288
	TB diagnosed	146
Northern Region	Total parishes	2628
	Total Sub-parishes	2654
	Sub-parishes screened	32
	Screening coverage	1.21%
	Total Individuals screened	6206
	TB diagnosed	115
Western Region	Total parishes	2872
	Total Sub-parishes	2915
	Sub-parishes screened	42
	Screening coverage	1.44%
	Total Individuals screened	5611
	TB diagnosed	93

**Table 3:** Distribution of number and proportion of hotspots and non-hotspots and their ACF coverage across the country, by region.

Region	Total Hotspots	Total Non-Hotspots	Total Sub-Parishes	Proportion of Hotspots	Proportion of Non-Hotspots
Central	954	1004	1958	48.7%	51.3%
Eastern	822	2804	3626	22.7%	77.3%
Northern	320	2334	2654	12.1%	87.9%
Western	1249	1666	2915	42.8%	57.2%

**Table 4:** Chest X-ray-based ACF yield in predicted hotspots and non-hotspots across the country.

Region	Hotspots	Non-Hotspots	Risk Ratio	95% CI (Risk Ratio)	Relative Risk Increase	Fischer’s Exact *p*-Value
Country	TB positive: 215	TB positive: 250	1.69	1.41–2.02	0.69	<0.001
	TB negative: 11,064	TB negative: 21,898				
Central	TB positive: 18	TB positive: 93	2.18	1.32–3.60	1.18	0.004
	TB negative: 905	TB negative: 10,306				
Eastern	TB positive: 75	TB positive: 71	1.52	1.10–2.10	0.52	0.011
	TB negative: 4140	TB negative: 6002				
Northern	TB positive: 49	TB positive: 66	1.74	1.21–2.51	0.74	0.003
	TB negative: 1805	TB negative: 4286				
Western	TB positive: 54	TB positive: 39	2.04	1.35–3.06	1.04	<0.001
	TB negative: 2217	TB negative: 3301				

## Data Availability

The raw data from community-based screening interventions used to train the model is the property of the National TB and Leprosy Control Programme, Ministry of Health Uganda. This data has restrictions on sharing due to ethical concerns. Requests to access these datasets should be directed to the same authority. The data on predictor variables like population and sociodemographic context was sourced from open platforms, and links to these data sources are included in the paper; they can be sourced easily. The final modelling outputs that were produced as results visualized in this paper can be made available in a repository.

## Abbreviations

ACF	Active Case-Finding
AI	Artificial Intelligence
CSO	Civil Society Organizations
CPD	Conditional Probability Distribution
DHS	Demographic and Health Survey
DCXR	Digital Chest X-Ray
GIS	Geographic Information System
GRDI	Gridded Relative Wealth Index
IDI	Infectious Disease Institute
LPHS-TB	Local Partner Health Services for Tuberculosis
MoH	Ministry of Health
NTLP	National TB and Leprosy Control Program
TB	Tuberculosis
USG	United States Government
VHTs	Village Health Teams
WHO	World Health Organization

## Appendix A

**Table A1:** Epidemiological variables and sources.

Variable Name	Definition/Description	Source	Resolution	Year
Overall population density	Number of people per square kilometre	Facebook	30 m	2016
Elderly (ages 60+) population	Counts of people over the age of 60 per square kilometre			
Female population	Counts of females per square kilometre			
Male population	Counts of males per square kilometre			
Distance to major roads	Distance from OpenStreetMap major roads to the centroid of a population cluster, measured in kilometres	WorldPop	3 arc s/~100 m at the equator	2018
DPT 1 vaccination received	Percentage of children 12–23 months who had received DPT 1 vaccination	Uganda Bureau of Statistics (UBOS) and ICF	5 × 5 km	2018
DPT 3 vaccination received	Percentage of children 12–23 months who had received DPT 3 vaccination			
Fully vaccinated (8 basic antigens)	Percentage of children 12–23 months who were fully vaccinated with 8 basic antigens (BCG, Polio 1–3, DPT 1–3, Measles)			
Measles vaccination received	Percentage of children 12–23 months who had received Measles vaccination			
Prevalence of stunting among children under 5 years of age	Percentage of children stunted (below −2 SD of height for age according to the WHO standard)			
Literacy men and women	Mean years of educational attainment in men (ages 15–49) and women (ages 15–49)	Institute For Health Metrics and Evaluation	5 × 5 km	2019
Prevalence of underweight among children younger than 5 years of age	Percentage of children under the age of 5 with weight-for-age Z-score (WAZ) less than −2 standard deviations from the WHO Child Growth Standards	Institute For Health Metrics and Evaluation	5 × 5 km	2019
Under 5 (0–5 years old) mortality probability	Probability of death for children under 5—mean estimates.	Institute for Health Metrics and Evaluation	5 × 5 km	2019
Access to any improved water source	Percentage of the de jure population living in households whose main source of drinking water is an improved source	Institute for Health Metrics and Evaluation	5 × 5 km	2019
Reliance on open defecation	Percentage of the de jure population living in households whose main type of toilet facility is no facility (open defecation)	Institute for Health Metrics and Evaluation	5 × 5 km	2019
HIV prevalence	Estimated prevalence among individuals aged 15–59 years	Institute for Health Metrics and Evaluation	5 × 5 km	2019
Elevation	Elevation above sea level (in metres)	WorldPop	100 m	
Motorized travel time to health facility	Optimal travel time to healthcare with access to motorized transport	Malaria Atlas	1 km	
Nightlights [nanoWatts/cm^2^/sr]	VIIRS data measured in nanoWatts/cm^2^/sr	Earth Observation Group	100 m	
Relative deprivation index	Composite socioeconomic deprivation index (2010–2020 average)	NASA Socioeconomic Data and Applications Center	1 km	

## References

[1] WHO (2023). WHO Conducts Mid-Term Review of Uganda’s Response to TB|WHO|Regional Office for Africa [Internet]. https://www.afro.who.int/countries/uganda/news/who-conducts-mid-term-review-ugandas-response-tb.

[2] Aceng F.L., Kabwama S.N., Ario A.R., Etwom A., Turyahabwe S., Mugabe F.R. (2024). Spatial distribution and temporal trends of tuberculosis case notifications, Uganda: A ten-year retrospective analysis (2013–2022). BMC Infect. Dis..

[3] Ho J., Fox G.J., Marais B.J. (2016). Passive case finding for tuberculosis is not enough. Int. J. Mycobacteriology.

[4] Henry N.J., Zawedde-Muyanja S., Majwala R.K., Turyahabwe S., Barnabas R.V., Reiner R.C., Moore C., Ross J. (2024). Mapping TB incidence across districts in Uganda to inform health program activities. IJTLD Open.

[5] Ayabina D.V., Gomes M.G.M., Nguyen N.V., Vo L., Shreshta S., Thapa A., Codlin A.J., Mishra G., Caws M. (2021). The impact of active case finding on transmission dynamics of tuberculosis: A modelling study. PLoS ONE.

[6] Sekandi J.N., List J., Luzze H., Yin X.P., Dobbin K., Corso P.S., Oloya J., Okwera A., Whalen C.C. (2014). Yield of undetected tuberculosis and human immunodeficiency virus coinfection from active case finding in urban Uganda. Int. J. Tuberc. Lung Dis. Off. J. Int. Union. Tuberc. Lung Dis..

[7] Robsky K.O., Kitonsa P.J., Mukiibi J., Nakasolya O., Isooba D., Nalutaaya A., Salvatore P.P., Kendall E.A., Katamba A., Dowdy D. (2020). Spatial distribution of people diagnosed with tuberculosis through routine and active case finding: A community-based study in Kampala, Uganda. Infect. Dis. Poverty.

[8] Kazibwe A., Twinomugisha F., Musaazi J., Nakaggwa F., Lukanga D., Aleu P., Kiyemba T., Nkolo A., Kirirabwa N.S., Lopez D.B.F. (2021). Comparative yield of different active TB case finding interventions in a large urban TB project in central Uganda: A descriptive study. Afr. Health Sci..

[9] Ochom E., Robsky K.O., Gupta A.J., Tamale A., Kungu J., Turimumahoro P., Nakasendwa S., Rwego I.B., Muttamba W., Joloba M. (2023). Geographic distribution and predictors of diagnostic delays among possible TB patients in Uganda. Public Health Action.

[10] Aturinde A., Farnaghi M., Pilesjö P., Mansourian A. (2019). Spatial analysis of HIV-TB co-clustering in Uganda. BMC Infect. Dis..

[11] Mergenthaler C., Mathewson J.D., Lako S., van der Merwe A.W., Potgieter M., Meurrens V., Latif A., Tahir H., Ahmed T., Samad Z. (2025). Predicting communities with high tuberculosis case-finding efficiency to optimise resource allocation in Pakistan: Comparing the performance of a negative binomial spatial lag model with a Bayesian machine-learning model. BMJ Public Health.

[12] Alege A., Hashmi S., Eneogu R., Meurrens V., Budts A.L., Pedro M., Daniel O., Idogho O., Ihesie A., Potgieter M.G. (2024). Effectiveness of Using AI-Driven Hotspot Mapping for Active Case Finding of Tuberculosis in Southwestern Nigeria. Trop. Med. Infect. Dis..

[13] Koura K.G., Hashmi S., Menon S., Gando H.G., Yamodo A.K., Budts A.L., Meurrens V., Lapelou S.-C.S.K., Mbitikon O.B., Potgieter M. (2025). Leveraging Artificial Intelligence to Predict Potential TB Hotspots at the Community Level in Bangui, Republic of Central Africa. Trop. Med. Infect. Dis..

[14] EPCON EPCON|Bayesian Network Approach [Internet]. https://www.epcon.ai/bayesiannetworkapproach.

[15] Rood E., Khan A.H., Modak P.K., Mergenthaler C., Van Gurp M., Blok L., Bakker M. (2019). A Spatial Analysis Framework to Monitor and Accelerate Progress towards SDG 3 to End TB in Bangladesh. ISPRS Int. J. Geo-Inf..

[16] John S., Abdulkarim S., Usman S., Rahman M.d.T., Creswell J. (2023). Comparing tuberculosis symptom screening to chest X-ray with artificial intelligence in an active case finding campaign in Northeast Nigeria. BMC Glob. Public Health.

[17] Babayi A.P., Odume B.B., Ogbudebe C.L., Chukwuogo O., Nwokoye N., Dim C.C., Useni S., Nongo D., Eneogu R., Chijioke-Akaniro O. (2023). Improving TB control: Efficiencies of case-finding interventions in Nigeria. Public Health Action.

[18] van Gurp M., Rood E., Fatima R., Joshi P., Verma S.C., Khan A.H., Blok L., Mergenthaler C., Bakker M.I. (2020). Finding gaps in TB notifications: Spatial analysis of geographical patterns of TB notifications, associations with TB program efforts and social determinants of TB risk in Bangladesh, Nepal and Pakistan. BMC Infect. Dis..

[19] Rahman M.S., Shiddik A.B. (2025). Utilizing artificial intelligence to predict and analyze socioeconomic, environmental, and healthcare factors driving tuberculosis globally. Sci. Rep..

[20] Facebook Connectivity Lab and Center for International Earth Science Information Network—CIESIN—Columbia University (2016). Global High Resolution Population Density Maps (Facebook Connectivity Lab, CIESIN)|UN-SPIDER Knowledge Portal [Internet]. High Resolution Settlement Layer (HRSL). https://www.un-spider.org/links-and-resources/data-sources/global-high-resolution-population-density-maps-facebook.

[21] WorldPop (2018). Global 100m Covariates [Internet].

[22] Uganda Bureau of Statistics (UBOS) and ICF (2018). Uganda Demographic and Health Survey 2016.

[23] Institute for Health Metrics and Evaluation (IHME) (2019). Low- and Middle-Income Country Educational Attainment Geospatial Estimates 2000–2017.

[24] Institute for Health Metrics and Evaluation (IHME) (2019). Global Under-5 Child Growth Failure Geospatial Estimates 2000–2019.

[25] Institute for Health Metrics and Evaluation [IHME] (2017). Low- and Middle-Income Country Neonatal, Infant, and Under-5 Mortality Geospatial Estimates 2000–2017 [Internet].

[26] (2020). Local Burden of Disease WaSH Collaborators. Mapping geographical inequalities in access to drinking water and sanitation facilities in low-income and middle-income countries, 2000–2017. Lancet Glob. Health.

[27] Dwyer-Lindgren L., Cork M.A., Sligar A., Steuben K.M., Wilson K.F., Provost N.R., Mayala B.K., VanderHeide J.D., Collison M.L., Hall J.B. (2019). Mapping HIV prevalence in sub-Saharan Africa between 2000 and 2017. Nature.

[28] VIIRS Nighttime Light. https://eogdata.mines.edu/products/vnl/.

[29] (2025). Global Gridded Relative Deprivation Index (GRDI).

[30] Malaria Atlas Project. Malaria Atlas Project. https://data.malariaatlas.org/trends?year=2024&metricGroup=Malaria&geographicLevel=admin0&metricSubcategory=Pf&metricType=rate&metricName=incidence.

[31] Ankan A., Textor J. (2024). pgmpy: A python toolkit for bayesian networks. J. Mach. Learn. Research..

[32] Data Tools and Practices. Institute for Health Metrics and Evaluation. https://www.healthdata.org/data-tools-practices.

[33] Maps and Regions—Office of the Vice President of Uganda. https://www.vicepresident.go.ug/maps-and-regions/.

[34] Mortazavi S.A., Swartwood N.A., Singh N., Can M.H., Cui H., Ryuk D.K., Horton K.C., Menzies N.A., MacPherson P. (2025). Urban and rural prevalence of tuberculosis in low- and middle-income countries: A systematic review and meta-analysis. medRxiv.

[35] Wynne A., Richter S., Banura L., Kipp W. (2014). Challenges in tuberculosis care in Western Uganda: Health care worker and patient perspectives. Int. J. Afr. Nurs. Sci..

[36] Johnson-Peretz J., Chamie G., Kakande E., Christian C., Kamya M.R., Akatukwasa C., Atwine F., Havlir D.V., Camlin C.S. (2023). Geographical, social, and political contexts of tuberculosis control and intervention, as reported by mid-level health managers in Uganda: ‘The activity around town’. Soc. Sci. Med..

[37] Seyedmehdi S.M., Jamaati H., Varahram M., Tabarsi P., Marjani M., Moniri A., Alizadeh N., Hassani S. (2024). Barriers and facilitators of tuberculosis treatment among immigrants: An integrative review. BMC Public Health.

